# Surgical Treatment of an Exuberant Case of Penoscrotal Elephantiasis

**DOI:** 10.7759/cureus.31470

**Published:** 2022-11-14

**Authors:** Frederico P Guerreiro, Ângelo Sá, Joaquim Seixas-Martins, Artur Canhoto

**Affiliations:** 1 Plastic, Reconstructive, and Maxillofacial Surgery, Centro Hospitalar de Lisboa Ocidental, Lisbon, PRT; 2 Plastic and Reconstructive Surgery, Centro Hospitalar de Lisboa Central, Lisbon, PRT; 3 Plastic and Reconstructive Surgery, Centro Hospitalar de Lisboa Ocidental, Lisbon, PRT; 4 Urology, Centro Hospitalar de Lisboa Ocidental, Lisbon, PRT

**Keywords:** obesity-related illnesses, scrotal edema, elephantiasis, management of lymphedema, primary lymphedema

## Abstract

Lymphedema is a manifestation of lymphatic system disturbance and deranged lymph transport, with resultant swelling, a proliferation of parenchymal and stromal elements, and excess deposition of the extracellular matrix. It may occur in any part of the body, most frequently in the limbs. Staging ranges from inconspicuous lymphatic system derangement to lymphatic elephantiasis. Surgical treatment is the preferred modality. This case report is of a 36-year-old male patient with morbid obesity with a five-year-long history of penoscrotal volume increase without any apparent trigger. Patient observation revealed a frankly enlarged scrotum involving the penis, with distortion and an increase in urinary meatus diameter. The penis was palpable but hardly observable. Neither testicle was palpable. Scrotal tissue was hardened and sclerotic. We performed surgical excision of all swollen tissue while identifying and preserving the penis and both testicles. Local advancement flaps were used to create a neoscrotum. Resurfacing of the penis was accomplished with split-thickness skin grafting harvested from a small part of healthy skin included in the excised tissue and held in place during the first week with negative pressure therapy. There are no signs of local or distant relapse, and the patient mentions a dramatic improvement in urinary flow, quality of life in terms of ambulation, everyday tasks, and self-esteem. We present a very rare clinical case of exuberant penoscrotal lymphedema in a young patient with very few risk factors. Given the extent and time of presentation, microsurgery of the lymphatics was not indicated, and thus, a Charles procedure was undertaken. Even so, patient quality of life was significantly improved, and no recurrences have been reported so far.

## Introduction

Lymphedema is an external and/or internal manifestation of a lymphatic system disturbance and deranged lymph transport. It may be classified as acute, transitory, or chronic depending on the duration of presentation and either primary or secondary based on its etiology. Regardless, the common denominator is that lymphatic system transport no longer has the capacity needed to handle a load of microvascular filtrate (either from lymphatic system insufficiency or excess production of volume), thus producing swelling. In response, there is a proliferation of parenchymal and stromal elements, with excess deposition of extracellular matrix substances and adipose tissue. Lymphedema is also classified according to its mechanism, low output or high output, and their respective etiologic agents (Appendices) [1].

In its most frequent form (i.e., peripheral lymphedema of the limbs), it often becomes a chronic, incurable condition, which, without proper management, may lead to repeated infections (cellulitis and lymphangitis), trophic skin changes, crippling invalidism, or, rarely, the development of Stewart-Treves syndrome (a highly lethal lymphangiosarcoma) [1].

Aside from the known conditions that may lead to lymphedema, there are known risk factors, such as body mass index (BMI) > 25 kg/m^2^, axillary node dissection, radiation to the axilla, and postsurgical cellulitis [1].

According to the International Society of Lymphology (ISL), the staging of lymphedema is made with a four-stage scale, starting with stage 0 (or Ia), referring to a condition where swelling is not yet observed, but subtle alterations already exist. Further stages already refer to overt edema. Stage I represents the early accumulation of high protein-containing fluid with the possibility of pitting. In stage II, pitting manifests, and limb elevation no longer reduces swelling. Excess fat deposition and fibrosis develop. Stage III refers to lymphatic elephantiasis, where pitting is absent with acanthosis, skin thickening, more fat and fibrosis deposition, and warty overgrowths instead. This staging system only encompasses the physical appearance of the limb [1].

The diagnosis of this condition is usually determined from clinical history and physical examination. Nevertheless, imaging studies such as ultrasound or, in specialized centers, lymphangioscintigraphy (LAS), among others, may have a role in clarifying less obvious cases or further classifying the condition. Genetic testing has become increasingly available and practical and may also be useful in screening specific syndromes [1].

Treatment is divided into non-operative and operative methods. Among the first group, physical therapy and adjuvants (such as elastic compressive garments), drug therapy directed against infection and the accumulation of fluid, and psychosocial rehabilitation are the main alternatives [2]. Operative approaches may include lymphatic vessel reconstruction via microsurgical or supramicrosurgical techniques or surgical resection of the affected skin and subcutaneous tissue. This latter approach is especially indicated in the most severe forms of fibrosclerotic lymphedema (also known as elephantiasis) [1,3-7].

## Case presentation

We present the case of a 36-year-old male with a known medical history of mild oligophrenia of unknown etiology, morbid obesity (with BMI of 51 kg/m^2^), type 2 diabetes mellitus, arterial hypertension, and sleep apnea/hypopnea syndrome.

This patient presented himself to our outpatient clinic with complaints of significant scrotal volume increase for five years with no previously associated trauma, neoplasm, medical/surgical treatment, or travels out of the country. According to the patient, this mass implied significant difficulties with ambulation and a decrease in urinary flow strength.

**Figure 1:**
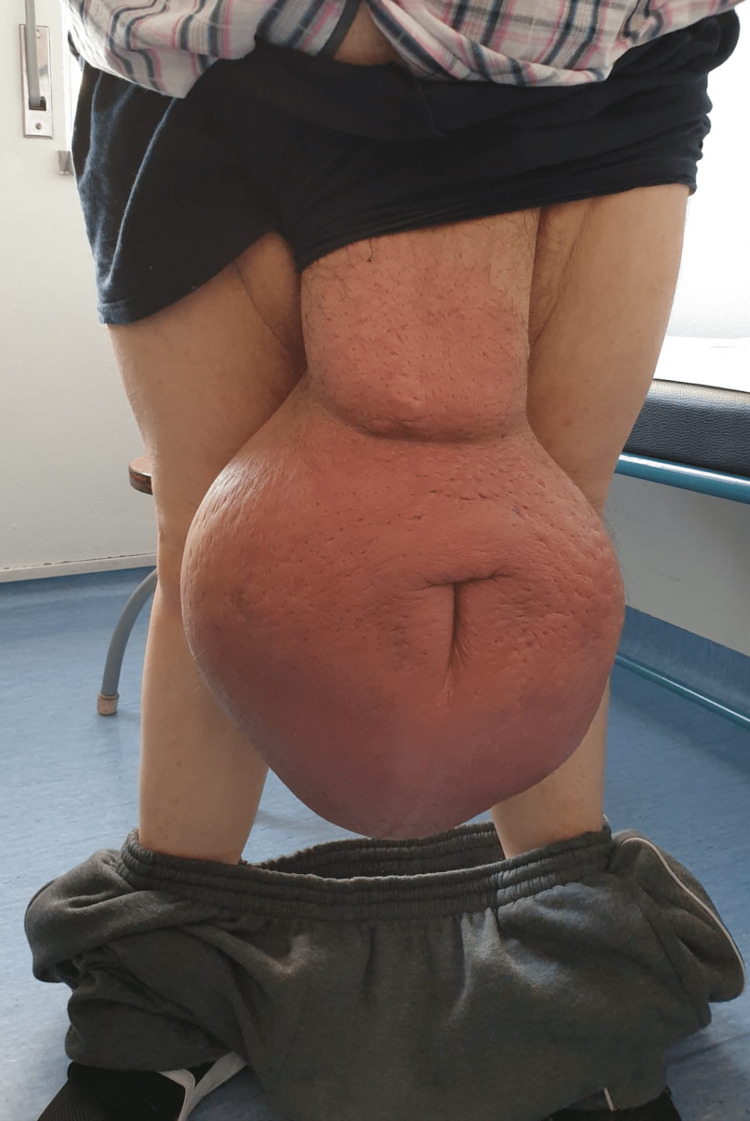
Patient presentation in our outpatient clinic

Patient observation revealed a frankly enlarged scrotum involving the penis, with distortion and an increase in urinary meatus diameter. The penis was palpable but hardly observable. Neither testicle was palpable. Scrotal tissue was hardened and sclerotic, with scarce inflammatory signs (Figure 1).

Complimentary studies included ultrasound (US) and computed tomography (CT), revealing radiologic findings compatible with the thickening of scrotal layers suggesting lymphedema. Both testicles and penis appeared to be structurally intact. No hernias of any kind were identified. Blood was tested for *Wuchereria bancrofti *antigen, which was negative. Examination of blood smears was also performed, with no relevant findings. Antifilarial antibody testing was not performed.

**Figure 2:**
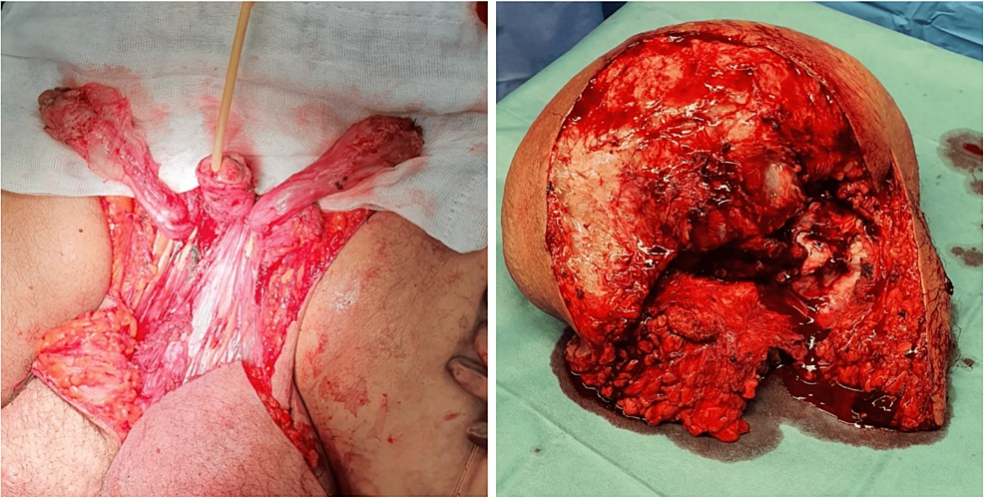
Operative procedure The left picture shows the patient immediately following the excision of diseased tissue, with evidence of structural integrity of both testicles and penis. The right picture shows the excised mass, weighing nearly 10 kg.

**Figure 3:**
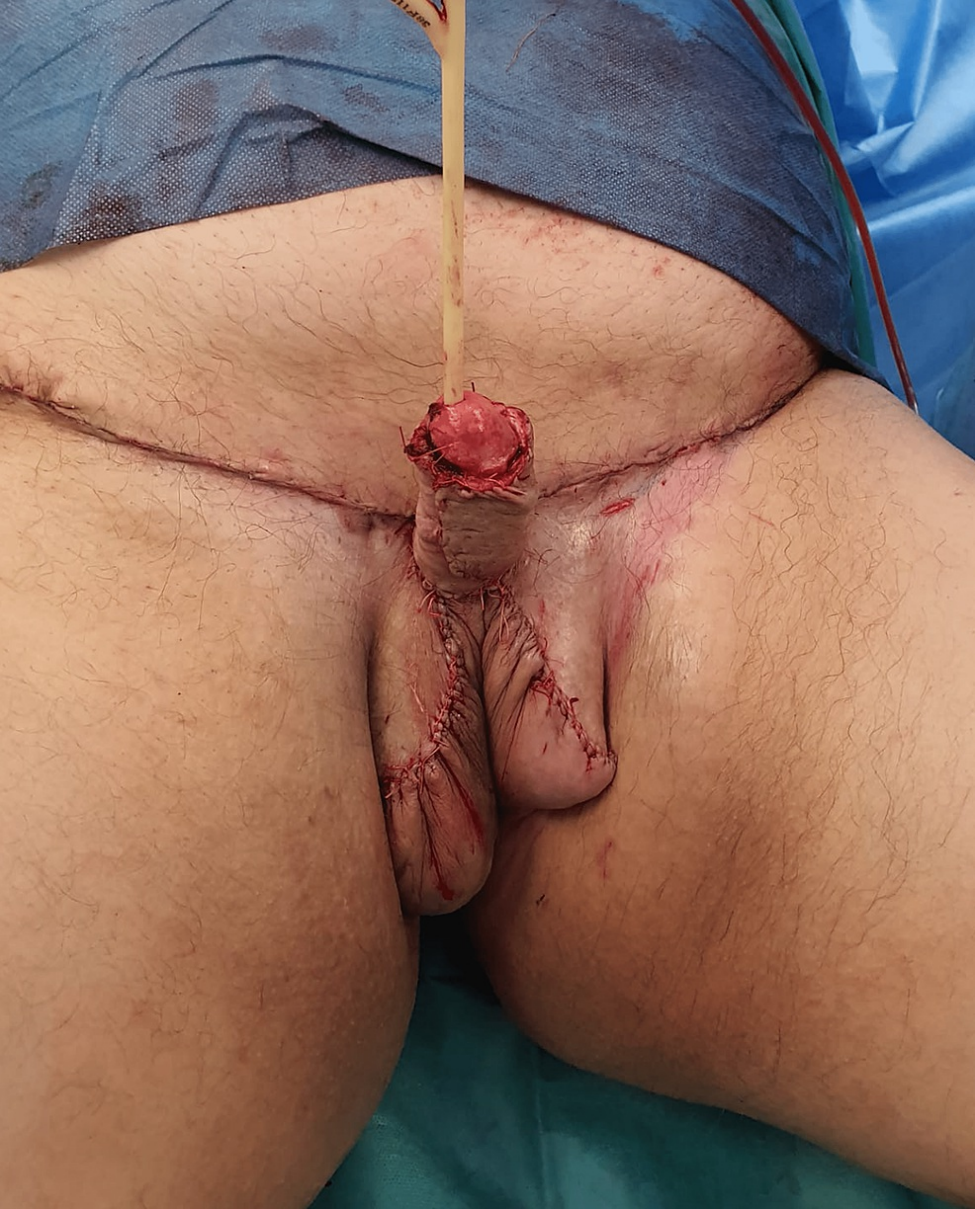
Immediate postoperative results showing the testicles inside the neoscrotum and the penis resurfaced with a split-thickness skin graft

The patient thus underwent surgical treatment, given the advanced stage of the disease, in a collaboration between the Plastic Surgery and Urology surgical teams. We performed surgical excision of all swollen tissue while identifying and preserving the penis and both testicles (Figure 2). The resulting defect was reconstructed with local advancement flaps, which were used to create a neoscrotum. Resurfacing of the penis was accomplished with split-thickness skin grafting harvested from a small part of healthy skin included in the excised tissue and held in place during the first week with negative pressure therapy (Figure 3).

The patient healed without any occurrences, with a full take of the skin graft. The patient stayed in the Plastic Surgery nursery for a total of two weeks and was then discharged to our outpatient clinic.

The pathology report revealed extensive sclerosis and fibrosis throughout the excised tissue. Microfilariae and/or adult worms were not found in the tissue analysis. No other relevant findings were reported.

**Figure 4:**
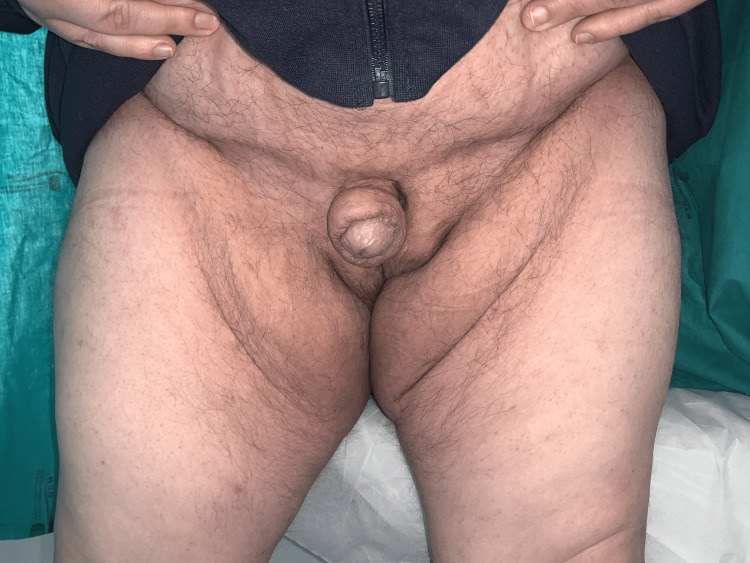
Two-year postoperative results

After two years postoperatively, there are no signs of local or distant relapse, and the patient mentions a dramatic improvement in urinary flow, quality of life in terms of ambulation, everyday tasks, and self-esteem. The patient also mentions great satisfaction with the cosmetic results achieved (Figure 4).

## Discussion

Penoscrotal lymphedema is a very rare condition in non-endemic regions, and a presentation such as the presented clinical case (lymphedema stage 3) is even more uncommon. Patient history revealed no significant risk factors for this condition other than obesity. Aside from the exuberant presentation, the patient mentioned very significant difficulties with the simplest aspects of everyday life, such as ambulation, wearing clothes, urinary voiding, or sexual intercourse. Also, such a mass posed a high risk of local infections (with potentially devastating consequences, given that the patient is a diabetic) because of mechanical stress and constraints with personal hygiene.

As mentioned above, surgical treatment of lymphedema may be directed to the correction of the lymphatic obstruction or excision of the affected tissue. In this case, trophic skin and penoscrotal tissue changes with fibrosis and sclerosis were so significant that the reestablishment of a normal lymph flow via microsurgical techniques was contraindicated. As in most other reported cases, we opted for radical surgical excision of the whole tissue. Given the need of preserving the external genitalia, we felt that collaboration with our Urology department was mandatory. In this procedure, we managed to excise the whole tissue surrounding the penis and both testes and safeguard their structural integrity, with exception of the penile skin. Tissue weighing approximately 10 kg was resected. Using local healthy tissues, advancement flaps were used to create a new scrotum and close the remaining defects. Using healthy skin excised together with diseased tissue, we managed to cover the penile shaft with a skin graft without the creation of another donor area.

Our surgical approach was like most other similar reports; also similarly, postoperative results were frankly positive [3-9]. To this date, all scars are maturing favorably with no signs of contracture or enlargement.

We consider that the patient was given a significant improvement in function, esthetics, and overall quality of life. It remains to be determined, however, if patient fertility was or was not affected by the five-year-long disease evolution.

## Conclusions

We present a very rare clinical case of exuberant penoscrotal lymphedema in a young patient with very few risk factors. Given the extent and time of presentation, microsurgery of the lymphatics was not indicated, and thus, a Charles procedure was undertaken. Even so, patient quality of life was significantly improved, and no recurrences have been reported so far.

## Appendices

Lymphedema is classified on the basis of its mechanism, low output or high output, and their respective etiologic agents (Table 1).

**Table 1:** Classification of lymphedema according to its mechanism

High output	Low output
Ascites	Congenital lymphatic dysplasia (primary lymphedema)
Anasarca	Anatomical obliteration (due to surgical resection, infection, or inflammation)
Right heart failure	Functional deficiency (spasm, stasis, and valvular insufficiency)
Deep venous insufficiency of the lower limb	
